# The importance of prognosis in geriatric patients attending the emergency department: a comparison between two common short geriatric assessment tools

**DOI:** 10.1007/s40520-023-02603-8

**Published:** 2023-11-06

**Authors:** Agnese Di Prazza, Baldassare Canino, Mario Barbagallo, Nicola Veronese

**Affiliations:** 1 Department of Health Promotion, Mother and Child Care, Internal Medicine and Medical Specialties, University of Palermo, Palermo, Italy; 2 Geriatric Unit, Department of Internal Medicine and Geriatrics, University of Palermo, Via del Vespro, 141, 90127 Palermo, Italy

**Keywords:** Prognosis, Multidimensional frailty, Emergency department, Mortality

## Abstract

**Background:**

The use of short geriatric tools in the emergency department (ED) is increasing, but the literature is still conflicting. The aim of this study is to compare the precision and the accuracy of two short geriatric assessment tools to predict mortality in a cohort of older patients attending the ED.

**Methods:**

A retrospective study was conducted including patients ≥ 65 years, attending the ED and transferred to a medical assessment unit from February to July 2022. Clinical Frailty Scale (CFS) and Brief Multidimensional Prognostic Index (Brief MPI) were administered. The association between Brief MPI and CFS and mortality was analysed via area under the curve (AUC) with its 95% confidence intervals (CIs), the C-statistics and a multivariate Cox’s regression analysis, in the latter case reporting the data as hazard ratios (HRs) with their 95% CI.

**Results:**

Among the 579 patients enrolled (mean age: 77 years), both Brief MPI and CFS showed a good accuracy in predicting mortality (AUC: 0.72; 95% CI: 0.61–0.83 for Brief MPI; 0.754; 95% CI: 0.65–0.83 for CFS). The discrimination of Brief MPI and CFS in predicting mortality was excellent, since the C-index of the Brief MPI was 0.85 and of CFS = 0.84. In the multivariate analysis, the risk for mortality was significantly increased for frailer subjects (HR 4.65; 95% CI: 1.45–15.00 for Brief MPI > 0.66; HR = 9.24; 95% CI: 1.16–76.90 for CFS > 6).

**Conclusions:**

Brief MPI and CFS showed a good accuracy/precision to predict mortality in older patients attending the ED. Considering that they are quick to perform, their introduction in ED clinical practice could be extremely helpful.

## Introduction

In the last years, there has been an increasing number of geriatric patients attending the emergency department (ED). By 2050, the world’s population of people aged 60 years and older will double. The number of persons aged 80 years or older is expected to triple between 2020 and 2050 to reach 426 million [1]. It is consequential that in the last 30 years, there has been a rise in older people attending the ED and this is shown in several studies conducted in different countries. In the UK, between 1990 and 2004, there was a 54% increase in total patients with a disproportionate 198% increase in patients aged more than 70 years, including a 671% increase in those aged more than 90 years [2]. In the USA, the population of people attending ED over 65 is expected to grow by 92%, those over 85 by 198%, and those over 100 by nearly 620%. Geriatric patients present to the ED with more severe conditions and require more resources during their ED visits compared with younger patients [3, 4]. In addition, compared with younger patients, the length of stay for older adults in the ED is significantly longer by 20% [5].

Emergency care units play an important role in healthcare providing interventions for acute and emergency issues in older people. Emergency physicians may not have expertise in treating older patients and may fail to identify relevant geriatric conditions such as dementia, depression, delirium, malnutrition. Older adults are also more likely to experience social isolation, malnutrition, and abuse or neglect, which may contribute to their ED presentation and influence outcomes [6]. A key element to consider when describing older patients is frailty. Frailty is a clinical condition of decreased physiologic reserve that leads to a vulnerable state and increases the risk of adverse health outcomes when exposed to a stressor in older adults. A review conducted by Kojima et al. highlighted that frailty is a significant predictor of ED utilisation among community-dwelling older adults [7]. Nearly 20% of older patients present to the ED with a specific self-care problem, such as those related to cognitive and functional impairments or difficulties with activities of daily living, despite increased levels of acuity, resource use, and higher need for hospitalisation in the older adult [8]. The traditional emergency model has been to focus on one problem per patient, whereas frail older adults sometimes require a more holistic approach, so many of these self-care issues are overlooked or otherwise not considered by ED clinicians who are focused on time-sensitive disease and injury [9]. At the same time, the role of CGA (comprehensive geriatric assessment) for the management of older patients in the ED is still poorly known, but it could be of importance since it captures the problems of frail older people.

Given this background, the primary focus of this study is to compare the accuracy and the precision of two common short CGA tools (i.e. Brief Multidimensional Prognostic Index [MPI] and the Clinical Frailty Scale [CFS]) in predicting mortality in older patients attending the ED.

## Materials and methods

### Study design and setting

This work is a single-centre retrospective study in which geriatric patients attending the ED and transferred to a medical assessment unit of the Policlinico Paolo Giaccone di Palermo between February 2022 and July 2022 were enrolled.

### Selection of participants

The inclusion criteria were age ≥ 65 years old patients, admitted to the ED and transferred to a medical assessment unit. The exclusion criteria were: age < 65 years old and patients who denied consent to receive Brief MPI and CFS. The medical assessment unit in our hospital provides a rapid definitive assessment, investigation and treatment for patients after ED and that need a longer hospital stay.

All data analysed here were collected as part of routine diagnosis and treatment. In agreement with the current Italian law (Gazzetta Ufficiale della Repubblica Italiana, Serie Generale n. 76 del 31-03-2008), we informed our local ethical committee of this observational research by sending a formal letter.

### Interventions

In this work, two tools were used for evaluating the presence of multidimensional frailty, i.e. Brief MPI and CFS. Brief MPI includes the evaluation of several domains important in older people including functional, cognitive, mobility, nutritional, comorbidities, number of drugs, and cohabitation status. It requires on average 5 min for its execution and the score is attributed with the same method as the classic version of MPI, i.e. with a weighted score between 0 and 1, higher scores reflecting higher risk of mortality [10].

The CFS was introduced to summarise the overall level of fitness or frailty of an older adult after they had been evaluated by an experienced clinician. In our study, we used the nine-point scale from fit to very severely frail and terminally ill. Taking little time to perform, CFS is useful in triage and higher scores have been shown to be predictive of adverse outcomes in patients evaluated in the emergency department [11].

### Measurements

We recorded the following data: age, sex, triage code based on severity (white, green, yellow, red), date and time of triage admission, date and time of ED admission, date and time of medical assessment unit admission, date and time of medical assessment unit discharge, discharge allocation (e.g. internal medicine, orthopaedics), main diagnosis at admission, and main diagnosis at discharge.

### Outcomes

For patients admitted to hospital, report of discharge date and mortality was obtained using administrative data. The maximum time to event considered was 30 days from ED admission.

### Analysis

Descriptive statistics for continuous variables (reported as means and standard deviations, SDs) and for categorical variables (number, percentages) were summarised using the independent Student’s *T *test and the Chi-square test, respectively, and comparing dead and alive patients during follow-up.

The association between Brief MPI and CFS and the outcomes of our interest was analysed using different methods. First, we assessed the accuracy of Brief MPI and CFS, including sex and age, in determining mortality using the area under the curve (AUC) with its 95% confidence intervals (CIs). Second, the discrimination/calibration was analysed using the C-statistics (Harrell’s C). For both these estimates, values between 0.9 and 1 are considered excellent, 0.8–0.9 optimal, 0.7–0.8 very good, 0.6–0.7 good, and 0.5–0.6 fair. Finally, the association between the exposure tools and mortality was assessed using a multivariate Cox’s regression analysis and reporting the data as hazard ratios (HRs) with their 95%CI. After checking the assumptions of Cox’s regression analysis, in these models, we included Brief MPI and CFS, alternatively, as main exposure, using less frail patients, according to CFS or Brief MPI, as reference. The data were right censored. As covariates, we included age and sex, since significantly associated with mortality in the univariate analyses in the case of age with a *p *value < 0.10 as thresholds and forcing sex, since it is a common demographic factor. The tolerance among factors was analysed using the variance inflation factor (VIF) with a cutoff of two as reason of exclusion, but no factor was excluded for this reason.

All analyses were conducted using the software STATA (version 14.2; StataCorp., College Station, TX) and SPSS 25.0.

## Results

### Characteristics of study subjects

In total, 579 patients aged 65 years and over attending the ED and transferred to the medical assessment unit from February 2022 to July 2022 were enrolled in this study. Altogether, 273 were women and 306 men; the mean age was 77 years (range: 65–97). The main cause of ED admission was dyspnoea (16.1%), followed by anaemia (11.6%) and abdominal pain (11.4%). Only two patients died in the medical assessment unit, one from cardiac arrest and the other from intestinal occlusion. Of the patients considered, 32.8% of patients were transferred to the internal medicine ward, 30.2% to a speciality ward, 9.7% to surgery wards, 13.5% were discharged to home, 9.2% to a speciality clinic, and 2.8% refused hospitalisation. Of the 400 patients admitted to hospital, 5.3% died during the 30-day follow-up period. The median stay in the ED was 46 h (range: 2–215) and the median length of stay in hospital was 7 days (range: 1–78 days).

As reported in Table 1, the difference in mean age between alive and dead patients was not statistically significant (*p* = 0.10) as well as sex (*p* = 0.89). Patients who later died (*n* = 74) were more frequently assigned to a red code compared to their counterparts (37.5% vs. 10.1%, *p* < 0.001). Patients who died were frailer, as expected, both using CFS and the Brief MPI (*p* < 0.001 for both comparisons). Considering the single domains of the Brief MPI, only two domains were not associated with mortality, namely number of drugs taken (*p* = 0.19) and CIRS [Cumulative Illness Rating Scale] (*p* = 0.13) that did not differ between dead and alive patients.

**Table 1:** Baseline characteristics by survival status

Parameter	Alive (*n* = 555)	Dead (*n* = 24)	*p* value
Age (mean, SD)	77.30 (± 7.347)	79.83 (± 8.365)	0.10
Females (%)	47.2%	45.8%	0.89
Red triage code (%)	10.1%	37.5%	< 0.001
ADL (mean, SD)	2.37 (± 1.01)	1.21 (± 1.35)	< 0.001
IADL (mean, SD)	1.50 (± 1.09)	0.63 (± 0.97)	< 0.001
SPMSQ (mean, SD)	0.77 (± 1.05)	1.88 (± 1.42)	< 0.001
Number of drugs (mean, SD)	5.77 (± 3.10)	4.92 (± 3.63)	0.19
MNA (mean, SD)	0.52 (± 0.80)	0.88 (± 0.90)	0.035
Barthel mobility (mean, SD)	1.75 (± 1.13)	0.63 (± 1.01)	< 0.001
CIRS (mean, SD)	2.59 (± 1.10)	2.25 (± 1.03)	0.13
Living alone (%)	9.7%	8.3%	0.006
Brief MPI (mean, SD)	0.38 (± 0.20)	0.57 (± 0.25)	< 0.001
Brief MPI < 0.33 (%)	46.7%	20.8%	
Brief MPI 0.33–0.66 (%)	40.2%	37.5%	
Brief MPI > 0.66 (%)	13.2%	41.7%	< 0.001
CFS (mean, SD)	4.71 (± 1.58)	6.17 (± 1.73)	< 0.001
CFS 0–3 (%)	22%	4.2%	
CFS 4–6 (%)	61.6%	41.7%	
CFS > 6	16.4%	54.2%	< 0.001

*SD* standard deviation, *ADL* activities of daily living, *IADL* instrumental activities of daily living, *SPMSQ* short portable mental status questionnaire, *MNA* mini nutritional assessment, *CIRS* cumulative illness rating scale, *MPI* Multidimensional Prognostic Index, *CFS* Clinical Frailty Scale

### Main results

**Fig. 1:**
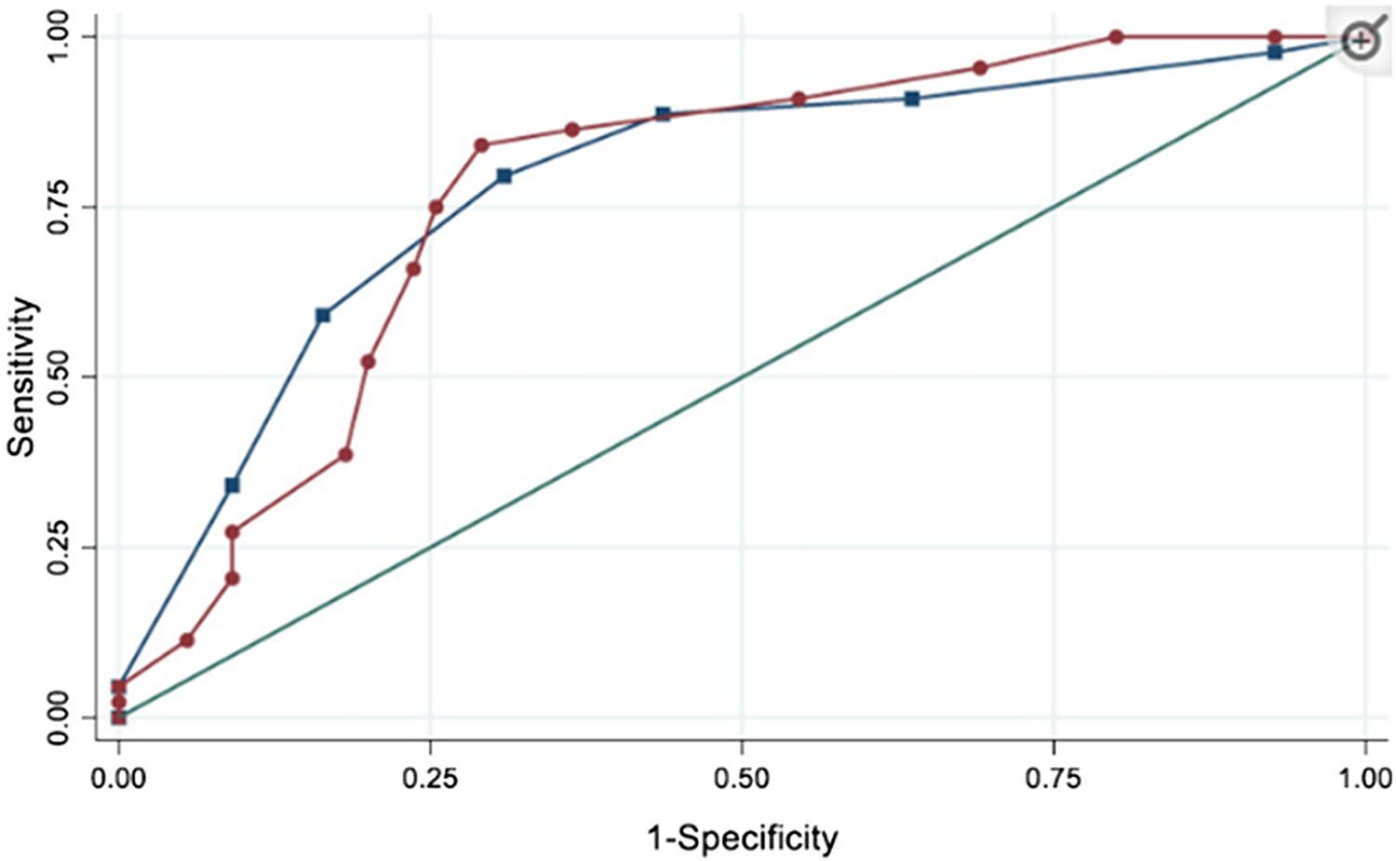
Accuracy of Brief Multidimensional Prognostic Index and Clinical Frailty Scale in predicting mortality. In blue Clinical Frailty Scale; in red Multidimensional Prognostic Index

Figure 1 shows the accuracy of both Brief MPI and CFS to predict mortality. The area under the curve is 0.72 (0.61–0.83) for Brief MPI and 0.754 (0.65–0.83) for CFS (*p* < 0.001). Regarding the Brief MPI, a value of 0.33 had a sensitivity of 79% and a specificity of 47%, while a value = 0.66 had a sensitivity of 42%, but a specificity of 87%. Similarly, a value of CFS of 3 was associated with a sensitivity of 96% and a specificity of 22%, while a value = 6 had a sensitivity of 54% and a specificity of 84%. The discrimination of Brief MPI and CFS in predicting mortality was excellent since the C-index of the Brief MPI was 0.85 and of CFS = 0.84.

As shown in Table 2, taking less frail as reference, in the multivariate analysis, the risk for mortality was statistically significant for frailer subjects (HR 4.65; 95%CI: 1.45–15.00 for Brief MPI > 0.66; HR = 9.24; 95%CI: 1.16–76.90 for CFS > 6, respectively) suggesting a strong association between frailty, assessed with different tools, and mortality.

**Table 2:** Association between Brief Multidimensional Prognostic Index and Clinical Frailty Scale and mortality

Parameter	Hazard ratios (95%CI)*	*P* value
Brief MPI < 0.33	1 reference	
Brief MPI 0.33–0.66	1.45 (0.47–4.50)	0.52
Brief MPI > 0.66	4.65 (1.45–15.00)	0.01
CFS 0–3	1 reference	
CFS 4–6	2.04 (0.25–16.67)	0.50
CFS > 6	9.24 (1.16–76.90)	0.036

*All analyses are adjusted for age and sex

The association between single domain of Brief MPI and mortality was also evaluated (Table 3). As the previous analysis, value = 0, indicating more robust patients for a prespecified domain, was taken as reference: when considering a value = 1, i.e. indicating a higher grade of compromission, the impairment of all domains resulted significantly associated with a higher risk of death, during the follow-up period.

**Table 3:** Association between single domains of the Brief MPI and mortality

Domain	Value = 0	Value = 0.5	Value = 1
ADL	1 reference	2.18 (0.56–8.55) *p* = 0.26	6.23 (2.39–16.24) *p* < 0.001
IADL	1 reference	0.69 (0.09–4.99) *p* = 0.71	3.80 (0.84–17.21) *p* = 0.082
SPMSQ	1 reference	0.65 (0.082–5.22) *p* = 0.68	16.18 (6.16–42.46) *p* < 0.001
Number of drugs	1 reference	0.36 (0.13–1.00) *p* = 0.05	0.32 (0.12–0.87) *p* = 0.026
MNA	1 reference	2.50 (0.97–6.41) *p* = 0.057	3.18 (1.15–8.77) *p* = 0.025
Barthel mobility	1 reference	2.52 (0.59–10.65) *p* = 0.20	8.69 (3.04–24.87) *p* < 0.001
CIRS	1 reference	0 *p*= 0.98	2.31 (1.00–5.29) *p* = 0.047
Cohabitation status	1 reference	2.82 (0.96–8.26) *p* = 0.058	0.77 (0.17–3.44) *p* = 0.73

*All analyses are adjusted for age and sex. All data are reported as hazard ratios with their 95% confidence intervals and their *p* values

## Discussion

The main aim of this work was to evaluate the validity and the accuracy of two short geriatric assessment tools to predict mortality and how frailty, assessed using two different tools and pathways, may influence outcomes in older patient in a delicate setting such as the ED.

It is well known that frailty is a condition frequently found in older people and it is associated with several adverse outcomes (such as disability, hospitalisation and finally mortality) also independently of other concomitant factors.[12] Our study confirms the epidemiological relevance of frailty, either assessed by CFS or Brief MPI, in ED. It is also important to highlight that the use of these tools makes the evaluation of frailty easier, being simple and cost-effective tools in comparison to other prognostic factors, which would require instrumental diagnostic elements or similar and, therefore, a longer time.

In our study, both Brief MPI and CFS were strongly associated with mortality showing a good precision and accuracy. In our opinion, this is a good result in relation to previous findings, as reported in a systematic review by Häseler-Ouart et al. that firstly described the use and the accuracy of CGA in ED [13]. In the aforementioned work, four studies which described the validity of geriatric assessment tools to predict mortality were included, the pooled sensitivity (95% CI) in short- and long-term mortality after ED admission was 0.77 (0.61–0.89) and 0.79 (0.46–0.96), and specificity (95% CI) was, respectively, 0.45 (0.32–0.59) and 0.37 (0.14–0.65) [13]. According to these findings, the assessment tools used in the studies included had a low predictive accuracy for mortality [13]. Overall, our study indicates that the choice to have two different points (one characterised by a higher sensitivity and one by a higher specificity) could be a good compromise from a clinical point of view in ED setting.

The main difference between the CFS and Brief MPI is that the first gives only one dimension of frailty, while Brief MPI and in general all versions of MPI provide a spectrum of impaired domains to act on. It is well known in literature that multidimensional frailty is more accurate in predicting adverse outcomes than other methods for diagnosing frailty [14, 15]. Performing a multidimensional assessment and the subsequent actions to correct the affected domains are achievable in settings like hospital wards or medical assessment unit, which was the object of this study. It could be difficult in an environment like the triage or the ED, because patients do not spend the necessary time and it could be time consuming, while patients may need to be stabilised. In this regard, our analyses showed that several domains are associated with an unfavourable outcome in older people attending the ED, suggesting that many aspects should be taken into account when assessing older patients. Given that Brief MPI and CFS are similar to predict mortality and it takes only few minutes to be performed, CFS, which relies only on medical judgement and is quicker than Brief MPI, could be performed in triage and ED in difficult clinical situations, while Brief MPI could be more suitable in medical assessment unit or when a better prognostic profile should be defined.

The findings of this work must be interpreted within its limitations. First, we used two short forms of CGA for which data regarding agreement with complete forms of CGA are still largely missing. Second, we did not assess the inherent changes of CFS and Brief MPI during the follow-up period and this could modify our findings. Third, the patients were evaluated in medical assessment unit that could introduce a selection bias, since, differently from ED, more severe patients are hosted. Finally, the CFS was executed using the admission data, whilst this scale should consider the level of frailty 2 weeks prior to presentation. However, from another point of view, we believe that our study indicates that the Brief MPI is possible to do to immediately understand the specific frailty-related factors present in the patients attending the ED.

In conclusion, Brief MPI and CFS are two short geriatric assessment tools with the purpose of giving a dimension of frailty in older people. In our study, both showed a great accuracy and precision to predict mortality in all patients regardless of age and sex. Considering that they are quick to perform, their introduction in ED clinical practice could be extremely helpful and future studies including the evaluation of frailty for clinical decision making could be useful.
